# Oncologist perspectives on the time toxicity of palliative systemic treatments for advanced cancer

**DOI:** 10.1093/jncics/pkaf111

**Published:** 2026-02-19

**Authors:** Samuel X Stevens, Isaac Y Addo, Ella El-Katateny, Brynna Rollins, Richard De Abreu Lourenço, Christopher M Booth, Joanne Shaw, Janette L Vardy

**Affiliations:** Faculty of Medicine and Health, The University of Sydney, Sydney, NSW, Australia; Department of Medical Oncology, Concord Repatriation General Hospital, Concord West, NSW, Australia; Department of Oncology, Queen’s University, Kingston, ON, Canada; Faculty of Medicine and Health, The University of Sydney, Sydney, NSW, Australia; Faculty of Medicine and Health, The University of Sydney, Sydney, NSW, Australia; Faculty of Medicine and Health, The University of Sydney, Sydney, NSW, Australia; Centre for Health Economics Research and Evaluation, University of Technology Sydney, Sydney, NSW, Australia; Department of Oncology, Queen’s University, Kingston, ON, Canada; Psycho-oncology Cooperative Research Group, School of Psychology, The University of Sydney, Sydney, NSW, Australia; Faculty of Medicine and Health, The University of Sydney, Sydney, NSW, Australia; Department of Medical Oncology, Concord Repatriation General Hospital, Concord West, NSW, Australia

## Abstract

**Background:**

People with advanced cancer often invest substantial amounts of time to receive palliative treatments. This has been labeled the “time toxicity” of cancer treatment. However, stakeholder views on time toxicity are still being established. This study used mixed methods to explore Australian oncologists’ perspectives on the time burdens of palliative systemic cancer treatments.

**Methods:**

Semistructured qualitative interviews were conducted with a convenience sample of gastrointestinal oncologists recruited from 1 metropolitan and 1 regional center, supplemented by online advertising through the Australian Gastro-Intestinal Trials Group. Themes emerging from initial interviews (*n* = 8) informed the development of an online survey disseminated to Australian oncologists via professional groups. Qualitative data were analyzed using an inductive approach. Survey data were summarized descriptively.

**Results:**

Fifteen oncologists were interviewed, 60% of whom were primarily based in major metropolitan areas. One overarching theme—the value of time—unified 4 subthemes: (1) contributors to time toxicity, (2) benefits and uncertainties, (3) time as a decision-modifier, and (4) proposed solutions. Surveyed oncologists (*n* = 108) expressed broad agreement with the thematic framework in interviews, affirming the importance of time for patients with advanced cancer and supporting strategies to reduce time burdens. However, responses acknowledged the subjectivity of time toxicity to individual patients.

**Conclusions:**

This mixed-methods study establishes Australian oncologists’ perspectives on the time toxicity of palliative systemic cancer treatments, identifying potential barriers and opportunities for including discussions of health-care time into shared decision making, and system-level strategies for addressing unwanted health-care contact time.

## Background

People living with advanced cancer often spend substantial time in contact with health care. Although health-care contact time (HCT) is essential for care, the concept of time as a hidden “toxicity” of treatment has contributed to discussions questioning the added value of some treatments.^1^ Patients may spend 10%-33% of their remaining life receiving health care, with HCT greatest at diagnosis and end of life.^2-9^ Although quantitative data on treatment time helps clinicians convey the time impact of care, qualitative studies are needed to understand the impact of HCT on stakeholders and clarify the value assumptions behind labelling time a treatment toxicity.

The imminent existential threat posed by a cancer diagnosis may heighten patients’ thresholds for enduring time burdens.^10^ Qualitative work suggests people with advanced cancer are hyperaware of the importance of time, with chronologic time burden from treatment activities intensified by uncertainties about the future.^11^ Time at home spent recovering from side-effects, coordinating treatment logistics, and handling financial and administrative demands also contributes to these burdens.^12-14^ Clinicians recognize these challenges and have expressed support for strategies to reduce them.^12^

Yet, oncologists’ perspectives on time toxicity remain underexplored. Two published studies have taken place in North America but may not be generalizable to other health-care contexts.^11^^,^^12^ Understanding oncologists’ perspectives on time toxicity is crucial given their role in influencing individual treatment decisions, research priorities, and health policy. This study aimed to comprehensively understand Australian oncologists’ perspectives on the time toxicity of palliative systemic cancer treatments using a mixed-methods approach.

## Methods

This mixed-methods study used semistructured interviews and an online survey. It was approved by Sydney Local Health District Human Research Ethics Committee (2023/ETH00869). Participants provided written informed consent and demographic data (gender, clinical role, years of experience, subspecialty, location, sector of practice) via Research Electronic Data Capture (REDCap) survey.^15^^,^^16^

### Qualitative study

Interviews were conducted between October 2023 and February 2025. A convenience sample of GI oncologists available to participate in interviews was recruited from 2 sites, supplemented by email and social media advertising distributed by the Australasian Gastro-Intestinal Trials Group and Friends of the Sydney Cancer Survivorship Centre. Gastrointestinal (GI) oncologists were selected due to high rates of HCT in this population.^3^^,^^4^ Participants were drawn from diverse locations to reflect geographic variability in care delivery and cancer outcomes.^17-19^

Interviews were conducted online or face-to-face using an interview guide informed by time and treatment burden literature.^11^^,^^20^ Open-ended questions facilitated rich discussion of the sources and consequences of HCT (Supplementary Methods). Interviews were audio-recorded, transcribed verbatim, and analyzed using thematic analysis tied to a framework approach in NVivo 14 (QSR International) (Table S1).^21^^,^^22^ Two researchers (S.S. and E.E.) developed a coding framework using an inductive, interpretivist approach based in grounded theory to anchor analysis in participants’ own language and framing of subjects discussed. Coding discrepancies were resolved through consensus with senior researchers (I.A. and J.S.) and the framework amended iteratively. Interviews continued until data saturation was reached. Reporting adhered to COREQ guidelines^23^ (Table S2).

### Oncologist survey

Themes from initial interviews (*n* = 8) informed development of a 28-item REDCap survey consisting of 5-point Likert responses, multiple-response, and open-ended questions (Table S3). The survey was piloted with 10 participants and refined according to feedback. It was advertised to Medical Oncology Group of Australia and Clinical Oncology Society of Australia members via email and social media and was open from August to November 2024. The target sample size was 100 responses, approximately 20% of the Australian medical oncology workforce.^24^ Survey data were analyzed using R version 4.4.2.^25^ Descriptive statistics were reported as medians and modes. Data were triangulated with qualitative findings to determine agreement with the thematic framework. Free-text responses were analyzed using the thematic framework derived from interview data.

## Results

### Qualitative interview analysis

Fifteen GI oncologists were interviewed (Table 1). One overarching theme, the value of time, unified 4 interrelated subthemes: (1) contributors to time toxicity, (2) benefits and uncertainties, (3) health-care time as a decision-modifier, and (4) proposed solutions. These themes were chosen from emergent codes as providing the most coherent insights into participants’ accounts of the research question. Illustrative quotes are referenced in text and in Table 2 by interview (Int), participant number and location (regional/metropolitan: R/M) (eg, Int-1-M: participant 1, metropolitan).

**Table 1.** Participant demographics, interview cohort.

Characteristic	Interview cohort, No. (%)	Survey cohort, No. (%)
**Gender**		
Female	7 (47)	Not asked
Male	8 (53)	
Non-binary	0 (0)	
**Primary role**		
Medical Oncologist	15 (100)	93 (86)
Medical Oncology Trainee	0 (0)	15 (14)
**Years of experience**		
<5 years	4 (27)	29 (27)
5-10 years	5 (33)	23 (21)
11-20 years	5 (33)	21 (19)
>20 years	1 (7)	20 (19)
**Tumor subspecialty** ^a^		
Breast	4 (27)	44 (41)
CNS	3 (20)	13 (12)
Genitourinary	5 (33)	31 (29)
Gynecological	4 (27)	18 (17)
General	5 (33)	25 (23)
Head and Neck	4 (27)	16 (15)
Lower Gastrointestinal	11 (73)	30 (28)
Lung	6 (40)	31 (29)
Melanoma/Skin Cancer	1 (7)	17 (16)
Sarcoma	2 (14)	9 (8)
Upper Gastrointestinal	12 (80)	29 (27)
Other	3 (20)	4 (4)
**Location of practice**		
Metropolitan	9 (60)	73 (68)
Regional	5 (33)	23 (21)
Both	1 (7)	9 (8)
**Sector of practice**		
Public	8 (53)	61 (56)
Private	0	10 (9)
Both	7 (47)	35 (32)

a All participants treated gastrointestinal malignancies, 10 participants reported that this was their dominant tumor subspecialty in interviews.

**Table 2.** Exemplar quotations from interviews.

Theme	Quotation
Value of time	“A lot is asked of patients with advanced cancer… We talk about improving quality of life so that they can live the life they want to live. On the one hand, we’re saying that, and on the other hand, we’re actively keeping them stuck in a whitewashed room with sick people, talking them about the thing that’s killing them.” (Int-20-R) “So I haven’t met anyone who … wouldn’t want to get out of the hospital or the hospital system. … No one wants to spend their time in hospital, especially when it’s so late in their life.” (Int-26-R) “I think it becomes more pronounced in people who are more borderline for treatment and more unwell and are fatigued by treatment whose, motivations and preferences and values move towards quality of life. I think that’s when they see the time toxicity as truly toxic.” (Int-25-M)
Contributors to “time toxicity”	“I do have some people who leave (remote town) at 3 or 4am, drive down, have chemotherapy, and then drive back on the same day, all before the (kanga)roos get out on the road. … That’s a rough day for them.” (Int-21-R) “I think that (physical) toxicity and time are interconnected. Because for me, time is not just time in the chair, but good quality time that you’re giving the patient and toxicity interacts with that.” (Int-65-M) “I kept her on the treatment, knowing that she was slowly progressing (whilst) she’s waiting for this trial treatment. She waited for 4 months for a slot on a phase one study.” (Int-58-R) “We don’t have the workforce to be able to provide the care that we want to for people. And it’s, you know, constantly double, triple booked in, you know … people just have to wait.” (Int-21-R)
Benefits of and uncertainties	“it is a toxicity, isn’t it? It’s a burden to the patient. An added issue that the patient has to deal with that they wouldn’t otherwise if we didn’t do the treatment we’re proposing. So I think it (the term ‘time toxicity’) is apt.” (Int-20-R) “I think what you’re getting at is quite a sophisticated, you know, philosophical assessment of what they’re going through. And with no disrespect to people who are in this situation, they are very overwhelmed by everything. Thinking really carefully about the things you’re thinking about might not be where their head is at.” (Int-58-R) “They’re a vulnerable patient population. They’re desperate. They’ve just been diagnosed with an advanced, incurable cancer. And most, but not all are willing to do ‘whatever it takes’, in order to survive. We do emphasize the quality of life. … But I don’t know that the time burden really comes into it.” (Int-25-M)
Time as a decision-modifier	“(For remote patients) I will probably put a little bit more emphasis on treatment that we can deliver through RVAC (Remote Video Accessed Chemotherapy) versus those that require a huge amount of travel, particularly when we are not going for curative treatment.” (Int-21-R) “if you’ve had an open discussion and you know there’s some evidence for it, it’s, you know, it’s hard to to say no sometimes.” (Int-65-M)
Proposed solutions	“With the complexity of sequencing and the rapidly evolving evidence, more time needs to be spent minimizing time toxicity for patients and discussing what really suits them the best. That is the society expectation. Versus (when) we had 20 minutes to see a patient 15 years ago when there was just FOLFOX and now there’s 5 things (and) more prognostic information, time toxicity, quality of life, the relative from overseas who wants to dial in, having to sort out someone’s camera. None of these things were happening before. … But the amount of time is the same. … That needs to change. Otherwise, we’re going to spend less and less time talking to people about what really matters to them.” (Int-56-M) “What actually we need, is like a patient navigator or care coordinator or cancer coordinator who can help patients and be along that journey with them. So there is a central point of contact of someone who is, um, capable, um, and understanding of the situation that the patients are in and who have dedicated time to help them navigate the healthcare system.” (Int-18-M) “(Healthcare time) is something we can actually fix, at least to a degree. And I think if we can recognize the things that we can fix, there will be a better pathway for those patients. Okay, well guess what? That’s exactly what we want for patients who have got advanced cancer.” (Int-19-M)

### Value of time

Interviews consistently reflected the perception that time held heightened intrinsic value for individuals with advanced GI cancers, with value proportional to prognosis: “The shorter your expectation of their life expectancy, the more important (time) becomes” (Int-19-M). However, participants had differing views on time spent in treatment. Most oncologists were circumspect about the benefits and potential burdens of time “lost” to treatment, particularly in second and subsequent treatment lines: “Though I can’t guarantee the outcome, I can guarantee hours of waiting round in a clinic … coming in for bloods” (Int-60-M). This manifested in conscious efforts to reduce treatment time where possible: “Do we really need the maintenance 5-FU?” (Int-56-M). In contrast, a minority of participants emphasized the need for urgent, effective treatment over concerns about time burden: “When someone is missing the boat and dying of metastatic disease … there is no quality. There’s no point talking about how life is because … they’re dying!” (Int-28-R).

### Contributors to time “toxicity”

Clinicians identified cumulative time receiving, commuting to, coordinating, or recovering from treatments as contributors to time burden. Perceptions of burden were felt to be highly patient dependent, relating to potential benefits of treatment, life stage, personal values, and patient’s competing priorities: “It’s actually the young patients that I think about more … those who have kids and have got to deal with family” (Int-53-M). Clinicians expressed frustration that patients’ time could be wasted when clinic time was spent clarifying administrative details, searching for results, or communications from other providers: “A lot of the initial interview establishing where or what investigations have been done” (Int-56-M). Considerable discussion was also dedicated to the impact of resource strain on wait times: “It’s just not possible, even with a registrar, to see 2 patients every 20 minutes” (Int-58-R).

Certain groups were perceived to experience a greater burden of HCT. Rural and remote patients often spent substantially longer travelling or away from home due to treatment requirements. For example, 5-Fluorouracil infuser disconnections necessitated “staying in town for 3 to 4 days on a fortnightly basis, 3 to 4 hours from their home” (Int-27-R). Patients from culturally and linguistically diverse backgrounds were also perceived as facing increased time burdens due to wait times for interpreters and greater potential for distress due to misunderstandings, which created “so many barriers, even (for) something as simple as getting a CT scan.” (Int-63-M). Early phase trial participants were perceived to undergo a large volume of health-care contact for uncertain benefit: “The chance of that working … really less than 5%, in my opinion” (Int-65-M). However, some countered that limited clinical trial places contributed to time toxicity via overuse of ineffective standard therapies.

### Benefits and uncertainties

Overall, participants felt that providing information on HCT was a “vital part of the consent process” (Int-20-R), routinely discussed through the lens of treatment scheduling. Most discussed cycle-to-cycle time burdens, though one participant reported conveying the proportion “of days that you are free to enjoy life with your family … in good function” (Int-56-M) throughout the treatment course. Other oncologists highlighted practical challenges with discussing HCT in this way, such as unawareness of the HCT outside of scheduled appointments and difficulty predicting the impact of side-effects on individuals.

The utility of providing information about HCT was questioned by some participants, who felt that it was unrealistic to expect patients to make “complex, philosophical assessments” (Int-58-R) of the value of HCT when facing information overload and emotional overwhelm. They wondered whether discussions about HCT would hold normative force for patients who were “doing absolutely everything they can at any gain, even if it is poor quality” (Int-65-M). Consequently, participants reported that the “toxicity” of treatment time was commonly realized only once patients were established on treatment.

Some participants questioned whether notions of time toxicity might better reflect clinicians’ rather than patients’ values. They spoke of the tangible physical and psychosocial benefits of treatment and expressed trepidation about the uniform application of negative terminology (toxicity) to the use of patients’ time. Participants suggested value-neutral terms such as “time commitment” (Int-19-M) or capturing time toxicity “in the positive: time away from doctors, enjoying life” (Int-56-M). The value of providing hope was mentioned: one participant remarked that although they “personally would not have treatments that take a long time and do very little,” they did not focus treatment discussions on time burdens because “if I said that to people, I feel I would destroy them” (Int-58-R).

### Time as a decision-modifier

Evidence for survival benefits and potential for treatment toxicity were key drivers of oncologists’ treatment recommendations. HCT was described as a “modifier” of treatment choice, along with other factors such as financial costs, logistics, and patient preferences: “honestly, (time and logistics) are third on my list after efficacy and safety” (Int-20-R). The importance of HCT in treatment decisions was relative to expected treatment benefit, and patient factors such as frailty, geographical or social isolation, and competing priorities: “The frail elderly are going to say: ‘I don’t want to come here and sit in your chair for hours and hours for the sake of 2 months ….’ The 32 year old, with 2 young kids at school goes ‘I’m doing everything I can, even for 1 day’” (Int-58-R).

### Proposed solutions

Participants offered solutions to reducing time burdens, centered around 5 areas: (1) increased health sector resourcing, including staffing and government reimbursement; (2) decentralizing care via telehealth and home-based services; (3) improved information exchange between providers; (4) enhancing care coordination; and (5) leveraging private sector capacity. All proposed broad solutions (nodes) with specific examples (codes) and supporting quotations from participants are displayed in Table 3.

**Table 3.** Oncologist proposed solutions to time “toxicity.”

Node	Code	Exemplar quote
Increased clinical resourcing	Increased staff resources	“A lack of public sector resourcing of specialist positions. I think that’s one major, major one. And I think it’s important to put down on paper because there’s a, I think, a general feeling around at the moment that there are too many specialists and, you know, everyone can be fit in. And I by no means believe that’s true.” (Int-56-M)
	Increased capacity	“We operate like an airline where we’re overbooked every day of the week and we have to slot people in and out as we get cancellations and so on.” (Int-58-M)
	Changes to Medicare reimbursement items	“Why is it that a phone call once a year with the patient for 30 minutes discussing their (rare tumor) reimbursed at half the amount as a standard follow-up?… From my point of view, there’s an economic disincentive to provide quality care.” (Int-56-M)
De-centralized care	Telehealth technologies	“It has been very helpful in doing, pre-treatment reviews and facilitating follow up. Yeah, I think I think it’s made it a lot easier for patients.” (Int-20-R)
	Visiting oncology services to remote areas	“When we have a chemotherapy suite and a visiting medical oncologist … the number of people that you’d expect for the population almost … actually exceeds what we see in (regional hub).” (Int-21-R)
	Upskilling staff in remote areas to administer chemotherapy	“Now that (small hospital) can give oxaliplatin, (travel time) has had less of an impact. To have to travel that far and lose a huge portion of your day once every couple of weeks…..” (Int-21-R)
	De-centralized clinical trials	“There is a lot of work on decentralized trials/telehealth that can be used to reduce time tox, and care coordination required to reduce tox as much as possible (eg, treatment at home, remote monitoring devices, etc).” (Su-76-M)
Improving information exchange	Incentivizing information sharing between providers	“The current environment where the I.T. coordinators of all the silos have a strong disincentive to help anything because really their job’s at risk if anything leaks… they’re not personally held to account if their patients are not able to access things quickly.” (Int-56-M)
	Single digital patient records	“When people are treated at different hospitals … information is not available on the electronic medical record. That is really challenging. People seeing private physicians who aren’t linked to any hospital record is challenging … correspondence from those notes don’t always make it to me. Or if they do, it comes 2 months later, which is not entirely helpful. Um, there’s just no real transparency to the different services that are available. … Part of (understanding time toxicity) is having some transparency of what patients are actually going through. … I think in order to properly capture (treatment time), we need to have that full understanding.” (Int-18-M)
	Information provision to patients	“If one patient resource has all that information, including time for blood tests and, you know, potential frequency of scans and, and frequency of visits that might be helpful to have just on a single sheet of paper.” (Int-20-R)
Enhanced care coordination	Nurse coordinators-navigators	“I think what actually we need is like a patient navigator or care coordinator or cancer coordinator who can help patients and be along that journey with them. So there is a central point of contact of someone who is, capable, and understanding of the situation that the patients are in and who have dedicated time to help them navigate the healthcare system.” (Int-18-M)
	Proactively coordinating healthcare contacts	“So we try to do things like getting patients to do their, their blood tests. A couple of days before their treatments In their local town. And we give them the chemo suite number to call up to just check that their bloods are okay to come down just to save them the massive trip back and forth.” (Int-21-R)
Reallocating resources	Use of private sector resources to cope with demand	“We send most of our biopsies to the private (hospital). There’s a financial toxicity problem there, but not a time toxicity problem. And they’re pretty efficient.” (Int-55-M)
	Reconsidering treatments with uncertain benefits	“Do we really need the maintenance 5-FU?” (Int-56-M)

### Survey responses

**Figure 1.**
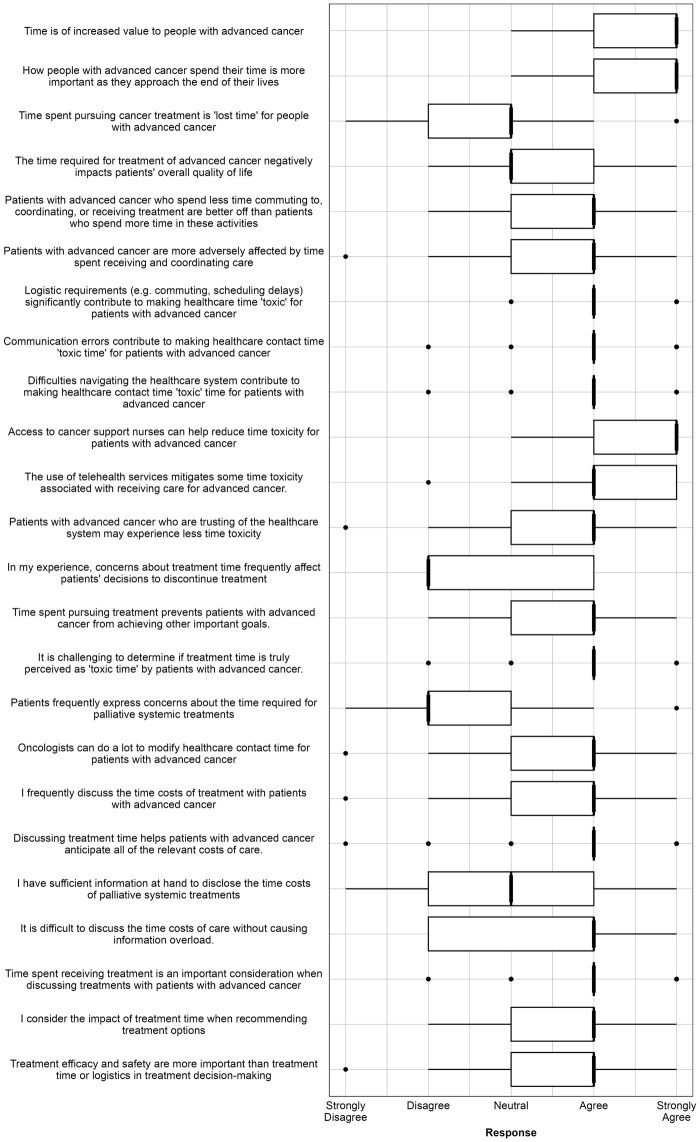
Oncologist perspectives on “time toxicity”: results of Likert questions.

There were 143 survey attempts with 108 completions (76%). Most respondents were medical oncologists (86%); 14% were trainees (Table 1). Likert responses are summarized in Figure 1; full results are available in Table S3. Trends are summarized as question number, median response value (eg, Q1; 5), with open-ended responses summarized by survey (Su), participant number, and rurality (eg, Su-1-M).

Oncologists strongly agreed that time was of increased value to people with advanced cancer (Q1; 5) and the value of time increased toward the end of life (Q2; 5), reflecting the view of interview participants that time was “of the essence, and at a premium” (Int-26-R) for patients with advanced GI cancers. Most agreed that patients who spent less time commuting to, receiving and coordinating care were better off than patients who spent more time in these activities (Q5; 4). However, 80% of participants did not agree that time spent pursuing cancer treatment was “lost time” (Q3; 3, mode = 2). This reflected the perception of interview participants that the toxicity of treatment time was subjective: “I’m not sure that patients really see it as a toxicity, at least initially” (Int-25-M). The magnitude of treatment benefit was emphasized as a determinant of HCT toxicity: “It is inversely proportional to how much benefit they feel they are receiving” (Su-7-M).

Survey respondents agreed it was challenging to determine when and if treatment time is truly perceived as toxic by patients (Q16; 4), reflecting interviewee’s uncertainty about the practical utility of information about treatment time to patients. Participants disagreed that patients frequently expressed concern about (Q17; 2), or discontinued treatment because of time burdens (Q13; 2). Strategies for observing time toxicity in clinical practice included oncologist-initiated strategies, eg, “conversations regarding values and goals of patients; regular discussions at critical points in treatment journey,” (Su-6-M) and/or relying on patient cues, for example, appointment nonattendance or patient-initiated goals of care discussions: “I rely on patients’ lived experiences—they usually tell me they have had enough” (Su-71-R).

Respondents consistently identified commuting to appointments (Q6; 4) and coordinating care (Q8; 4) as contributors to time toxicity. However, there was less consensus regarding whether time spent recovering from treatment-related side effects should be conceptualized similarly: “I’d distinguish time feeling (unwell) because of side-effects from the time needed for assessments, administration, travel ….” (Su-17-M). There was strong support for employing specialist nurse coordinators (Q10; 5) and using telehealth services (Q11; 5) to reduce the time impact of health care. Additional suggestions included streamlining administrative processes, expanding home care, and developing metrics to quantify time burden: “Working through ‘the system’ is a large factor in time toxicity … car parking, waiting times in overbooked clinics, chemo suites with limited capacity … telehealth and home-based treatment including chemo could ideally reduce patient time toxicity but sadly is likely to worsen the clinicians’ time toxicity” (Su-55-M).

Many participants endorsed using “time toxicity” as a prompt to reduce unnecessary care. Additional solutions are included in Table 3.

## Discussion

This mixed-methods study sought to understand Australian oncologists’ views on the time toxicity of palliative systemic treatments. Several important findings emerged. First, oncologists place considerable value on patients’ time, particularly when prognosis is poor. Second, translating HCT into subjective burden depends on patients’ perceptions of treatment benefits, downsides, and individual values. Third, HCT can be helpful in informing treatment decisions, though it is challenging to communicate this information to patients effectively and compassionately. Finally, several practical suggestions to reduce patient and clinician time burdens emerged. Agreement between survey and interview populations suggests that these findings may be generalizable to the broader Australian oncology community.

Our study contributes to the small but growing literature exploring perspectives on the time impacts of cancer care. Prior work affirms that patients with advanced cancer, their caregivers, and clinicians are acutely aware of the importance of time both as an abstract concept and limited resource.^11^ Time spent on treatment and its accompanying logistical requirements have previously been associated with a sense of burden, as well as disrupting activities unrelated to cancer care.^11-14^ This may include time spent on administrative and financial burdens associated with negotiating insurance approvals, taking leave from employment, and arranging social security payments to facilitate treatments.^10^^,^^12^^,^^13^

This study confirms many of these findings in an Australian context and extends knowledge of systemic contributors to unwanted HCT. Like patient and caregivers, participants in this study associated cumulative health-care contact with increased time burden.^12-14^^,^^26^ Compared with patients, fewer oncologists recognized the time impacts of care outside the hospital setting.^14^ In the context of a single-payer health system, time burdens related to financial and administrative activities were not a substantial focus.^12^^,^^13^ However, the exclusive focus on oncologist perspectives highlighted systemic contributors to unwanted health-care contact time in the Australian context. Challenges such as fragmented information exchange between providers, lack of shared health records, absence of patient-facing appointment management tools, and inadequate resourcing for growing consultation complexity represent actionable targets for quality improvement initiatives.

This work contrasts with prior studies of patient perspectives, revealing nuanced differences in how health-care time is perceived. Oncologists consistently framed patients’ time as a valuable commodity and were generally willing to modify treatment to reduce time burdens. Yet, many were uncertain about how patients themselves perceived these burdens, noting that time-related concerns were rarely raised in consultations. Temporal and logistical considerations were deprioritized unless prompted by patients or at key clinical milestones, such as disease progression. In contrast, Australian patients and caregivers recognized the opportunity costs of HCT but perceived they had little choice but to pursue palliative treatments to extend survival.^14^ Treatment time frequently interrupted patients’ ability to freely direct their lives, contributing to a disempowered sense that treatment resembled unpaid work. This supports Parson’s assertion that patients’ agency may be diminished due to the existential threat of a cancer diagnosis. The combination of patients diminished agency and oncologists’ deference to patient autonomy may create a barrier to meaningful discussions about the burdens of treatment.

These findings have important implications for patient-clinician communication and future research. Oncologists should be supported to incorporate discussion of treatment time burdens into shared decision making. Time spent in treatment is likely to be more understandable than technical details about treatment and may help restore patients’ sense of control, especially when contextualized with prognostic information. The structured communication framework proposed by Brown et al. offers a potential model for this approach.^27^ To support informed decision making, quantifying the time costs of care using real-world and prospective trial data is essential. Although there was conjecture about describing time recovering from side-effects in the same light as time in direct contact with health care, this was a prominent part of patients’ conception of time toxicity.^12-14^ Measures like Q-TWiST (Quality-adjusted Time Without Symptoms or Toxicity) provide an approach to describe the impact of treatment on the quality of time outside health care,^28^ but measures that assign weight for the combined burdens or toxicity of HCT are not yet available. When discussing treatment time with patients, the importance of patient-centric, value-neutral language was highlighted, for example, “time commitment.” However, participants acknowledged that the label “time toxicity” provokes reflection on potential interventions to reduce nonessential HCT. In addition to improved reporting, developing decision-making tools incorporating metrics of HCT could empower oncologists and supporting clinicians (eg, oncology nurse specialists) to assist patients with complex treatment decisions.

Several limitations should be acknowledged. First, the interview cohort was limited to GI oncologists practicing within a single-payer, resource-rich health system and may not be generalizable to other health systems. Second, the survey instrument was developed based on themes identified with a select group of oncologists. Third, broader insights might have emerged from including a wider range of oncology professionals such as nurses and social workers.

This mixed-methods study explored Australian oncologists’ perspectives on the time toxicity of palliative systemic cancer treatments and highlighted key differences between the perspectives of GI oncologists and the previously reported views of patients and caregivers.^14^ This layered understanding of the temporal impact of health care is essential for informing values-based shared decision making and addressing modifiable contributors to burdensome or unwanted health-care contact. By identifying what renders time “toxic” and clarifying the barriers and uncertainties surrounding discussions of health-care time in clinical practice, this work provides a basis for measuring and managing the time impacts of cancer care.

## Supplementary Material

pkaf111_Supplementary_Data

## Data Availability

The data underlying this article cannot be shared without compromising the privacy of individuals who participated in the study. The qualitative nature of the interviews and experiences of clinicians are personal. Even if identifying information is removed from individual transcripts, it may still breach confidentiality. Additional de-identified summary level data can be requested from the authors.
