# Supplementary material for: Oncologist perspectives on the time toxicity of palliative systemic treatments for advanced cancer
Source: JNCI Cancer Spectr. 2026 Feb 19;10(1):pkaf111. doi: 10.1093/jncics/pkaf111 (PMC12936397; doi:10.1093/jncics/pkaf111)
Supplement: pkaf111_Supplementary_Data [file pkaf111_supplementary_data.zip › Supplementary Materials.docx]

**Supplementary Materials**

**Supplementary Methods**

| Interview Guide: Gastrointestinal Oncologists |
| --- |
| - **For patients with metastatic disease, how do you think that a patient’s quality of life is impacted by the time needed to coordinate and receive treatments?**   *Time toxicity has been defined as the time that patients spend engaged with healthcare when they could be pursuing other activities.*   - **To what extent do you consider the time involved in accessing a treatment when prescribing palliative systemic cancer treatment to patients?**    - *Probes if yes: Are there groups of patients in whom you think this is more important? Why? (E.g. older age, poorer prognosis, remote geographic location, patient is a carer/has dependents).*   - *Probes if answer is no – Are other logistical considerations important?* - **What would you consider to be the most important factors when you are making a treatment recommendation?**    - *Probes: e.g. efficacy; physical side-effects; cost/financial toxicity; treatment logistics and convenience; personal and family responsibilities; attending important events or goals; impact on engaging in daily activities; emotional and cognitive side-effects; outcomes of not having treatment.* - **How often do you have explicit discussions about the time commitment involved with treatment?**    - *Probe: How do you frame these discussions?* - **If you had information at hand about the average time impact associated with different cancer treatments, how might you use it in clinical practice?**    - *Probe: How do you frame these discussions?* - **In your practice, what factors contribute the most to additional time burdens for patients whilst receiving treatment?** - **The term 'time toxicity' has been used to describe the impact of time associated with receiving cancer treatment. What are your reflections on this term?**   - *Probe: would you propose an alternative term?* - **Conclusion:** Thank you for agreeing to participate in this study. |

# Table S1: Framework Analysis

| Transcription | In this study, transcription was carried out by two authors (SS and EE). Transcription was initially performed automatically, using the online transcription service TRINT ([www.trint.com](http://www.trint.com)). SS and EE then examined transcripts for accuracy. We were primarily interested in the content of responses, thus, only long pauses, interruptions, non-verbal communication (laughter, crying) were noted within the text. Transcripts were checked for errors by peer review and clarification in regular research meetings. |
| --- | --- |
| Familiarisation | Four members of the research team reviewed initial transcripts (SS, EE, IA, JS) to refine the question guide and provide feedback on interview style. SS, IA and EE read and re-read all transcripts and listened to audio-recordings to become familiar with the whole dataset. Initial impressions were recorded using the annotation function in NVivo prior to coding. |
| Coding | Two members of the research team (SS, Medical Oncologist and PhD Candidate, and EE, Pharmacist and Medical Student) coded the same three transcripts in each cohort before meeting to discuss emergent codes. Codes consisted of short phrases to whole paragraphs describing a particular phenomenon or idea. We used a constant comparative method to ensure consistency of coding, with review by experienced qualitative researchers (IA, JS). |
| Developing a working analytical framework | SS and EE coded the same three transcripts in each cohort, then met to discuss emergent codes. An initial coding framework was developed in consultation with JS and JV. Disagreements about coding labels were discussed and consolidated, with input from a senior author if needed. A brief description was included in the codebook for consistency. Using this framework, a further three transcripts each were coded, before further meetings to revise and refine the framework. This process was repeated until no new themes were generated, and the final thematic framework was agreed. |
| Applying the analytical framework | We applied the final analytical framework to each transcript by importing transcripts in QSR NVivo version 14 for Mac and attaching appropriate codes from the analytical framework. All authors participated in organising codes into categories reflecting prominent themes within the data set. |
| Charting data into the framework matrix | A matrix was presented for each theme by abstracting, summarising and charting data for each case and each code within that theme. |
| Interpreting the data | Thematic analysis was carried out on the data set by reviewing matrices and making connections within and between codes and participants. This process allowed for explorations on how patients and caregivers within our dataset experienced the perceived and experienced the phenomenon of ‘time toxicity’. |

# Table S2: COREQ Checklist

| **Domain 1: Research Team and Reflexivity** | | | |
| --- | --- | --- | --- |
| **Personal Characteristics** | | | |
| 1 | Interview | Which author(s) conducted the interviews or focus group? | One researcher carried out interviews (SS). |
| 2 | Credentials | What were the researcher’s credentials? | SS is a PhD candidate, holds an MBBS (Hons) and is a Fellow of the Royal Australasian College of Physicians accredited to practice as a Medical Oncologist. |
| 3 | Occupation | What was their occupation at the time of the study? | SS is a medical oncologist and PhD Candidate employed at Concord Hospital. |
| 4 | Gender | Was the researcher male or female? | SS is male. |
| 5 | Experience and training | What experience or training did the researcher have? | SS has experience interviewing patients via his employment and was instructed on qualitative interviewing by JS and IA, who are experienced psycho-oncology researchers, including practical training. IA and JS provided additional indirect feedback via review of interview transcripts. |
|  | **Relationship with participants** | | |
| 6 | Relationship established | Was a relationship established prior to study commencement? | SS had worked professionally with some participants as a trainee or colleague in the same department. |
| 7 | Participant knowledge of the interviewer | What did the participants know about the researcher? | Three participants worked in the same department as the interviewer. Other participants were aware of where the researchers worked and purpose of the research. It was disclosed that this research would contribute to SS partially satisfying the requirements of a PhD.  Participants were self-selected and the existing relationships did not impact on participation.  None of the authors participated in interviews. |
| 8 | Interviewer characteristics | What characteristics were reported about the interviewer/facilitator? | The PIS declared that the interviewer was conducting this research to partially satisfy the requirements of a PhD. |
|  | **Domain 2: Study Design** | | |
|  | **Theoretical framework** | | |
| 9 | Methodological orientation and theory | What methodological orientation was stated to underpin the study? | Grounded theory was used. An inductive approach to analysis was taken, using thematic analysis, tied to a framework approach. A team-based, reflexive approach was used to coding, using the constant comparison method. |
|  | **Participant selection** | | |
| 10 | Sampling | How were participants selected? | A convenience sample of GI oncologists were recruited through participating sites, email and social media advertising with assistance from the Australasian Gastro-Intestinal Trials Group (AGITG) and Friends of the Sydney Cancer Survivorship Centre (FSCSC). The recruitment period was 5^th^ October 2023 to 7 February 2025. |
| 11 | Method of approach | How were participants approached? | This study also recruited patient and unpaid caregivers, who were referred to the study by their oncologists. Due to differing thematic content, the results of patient/caregiver interviews are presented elsewhere. Oncologists at participating sites for the patient/caregiver study were informed of the study via departmental presentation and email invitation. If they wished to participate in the study themselves, they were required to approach a member of the research team.  Other oncologist interview participants were recruited via email and social media advertising through the AGITG and FSCSC social media and email newsletters. |
| 12 | Sample Size | How many participants were in the study? | The *a priori* sample size was 10 participants. Thematic saturation was indicated after 8 participants. A further 7 participants were interviewed to achieve a total of 15 participants. |
| 13 | Non-participation | How many people refused to participate or dropped out? Reasons | Of 16 oncologists who approached the research team to participate, one oncologist was unable to participate due to scheduling issues and subsequent extended leave. |
|  | **Setting** | | |
| 14 | Setting of data collection | Where was the data collection? | Face-to-face interviews were conducted in a private clinical setting; online-interviews were conducted using Microsoft Teams (NSW Health License). |
| 15 | Presence of non-participants | Was anyone else present besides the participants and researchers? | Only the researcher and participants were present. |
| 16 | Description of sample | What are the important characteristics of the sample? | Important demographic information is summarised in the manuscript (Table 1). |
|  | **Data collection** | | |
| 17 | Interview guide | Were questions, prompts, guides provided by the authors? Was it pilot tested? | Semi-structured interview guides were developed by the researchers in consultation with a patient advocate and used during interviews. These were pilot tested in the initial interviews and amended iteratively. No major revisions were required. |
| 18 | Repeat interviews | Were repeat interviews carried out? | No repeat interviews were required. |
| 19 | Audio/visual recording | Did the research use audio or visual recording to collect the data? | Interviews were audio recorded using Microsoft Teams. Visual data automatically captured during recording was deleted by the researcher prior to transcription. |
| 20 | Field notes | Were field notes made during and/or after the interview? | No field notes were routinely collected. |
| 21 | Duration | What was the duration of the interviews? | Participants were advised that interviews would take up to 45 minutes. At 45 minutes, the interviewee was informed of the time and verbal consent was required to continue.  Interviews ranged from 22 to 58 minutes. |
| 22 | Data saturation | Was data saturation discussed? | Transcripts were reviewed and coded using the constant-comparative method. A teams based reflexive approach was used to continually assess for data saturation.  After 8 participants were recruited, thematic saturation was reached. A further 7 interviews were conducted to confirm thematic saturation. |
| 23 | Transcripts returned | Were transcripts returned to participants for comment and/or correction? | To ensure fidelity to the participants’ original impressions, transcripts were not returned for review. |
|  | **Data analysis** | | |
| 24 | Number of data coders | How many data coders coded the data? | Two researchers (SS and EE) coded transcripts independently, as described in the manuscript. Coding schemas were refined iteratively in consultation with IA and JS. |
| 25 | Description of the coding tree | Did authors provide a description of the coding tree? | A coding tree is available on request from the authors. |
| 26 | Derivation of themes | Were themes identified in advanced or derived from the data? | Themes were derived from the data. |
| 27 | Software | What software, if applicable, was used to manage the data? | NVivo 14 for Mac, licensed to the University of Sydney. |
| 28 | Participant checking? | Did participants provide feedback on the findings? | No |
|  | **Reporting** | | |
| 29 | Quotations presented | Were participant quotations present to illustrate the themes/findings? Was each quotation identified? | Quotations have been presented throughout the manuscript. |
| 30 | Data and findings consistent | Was there consistency between the data presented and the findings? | We endeavoured to report the study findings in a clear consistent manner to accurately reflect the data that have been collected. |
| 31 | Clarity of major themes | Were major themes clearly presented in the findings? | Yes, major themes are presented in the manuscript. |
| 32 | Clarity of minor themes | Is there a description of diverse cases or discussion of minor themes? | All data relating to the development of coding is presented in the manuscript. |

# Table S3: Australian Oncologist Perspectives on Time Toxicity of Palliative Systemic Cancer Treatments: Full Survey Results

| **Q** | **Question** | Strongly disagree (1) | Disagree (2) | Neutral  (3) | Agree  (4) | Strongly Agree  (5) | Median |
| --- | --- | --- | --- | --- | --- | --- | --- |
| **1** | Time is of increased value to people with advanced cancer | **0** | **0** | **3** | **42** | **55** | **5** |
| **2** | How people with advanced cancer spend their time is more important as they approach the end of their lives | **0** | **0** | **1** | **40** | **59** | **5** |
| **3** | Time spent pursuing cancer treatment is ‘lost time’ for people with advanced cancer | **2** | **42** | **36** | **17** | 3 | **3** |
| **4** | The time required for treatment of advanced cancer negatively impacts patients’ overall quality of life | **0** | **15** | **40** | **39** | **6** | **3** |
| **5** | Patients with advanced cancer who spend less time commuting to, coordinating, or receiving treatment are better off than patients who spend more time in these activities | **0** | **5** | **25** | **46** | **24** | **4** |
| **6** | Patients with advanced cancer are more adversely affected by time spent receiving and coordinating care | **1** | **10** | **23** | **60** | **6** | **4** |
| **7** | Logistic requirements (e.g. commuting, scheduling delays) significantly contribute to making healthcare time ‘toxic’ for patients with advanced cancer | **0** | **0** | **5** | **78** | **17** | **4** |
| **8** | Communication errors contribute to making healthcare contact time ‘toxic time’ for patients with advanced cancer | **0** | **6** | **14** | **63** | **17** | **4** |
| **9** | Difficulties navigating the healthcare system contribute to making healthcare contact time ‘toxic’ time for patients with advanced cancer | **0** | **2** | **4** | **72** | **22** | **4** |
| **10** | Access to cancer support nurses can help reduce time toxicity for patients with advanced cancer. | **0** | **0** | **5** | **41** | **54** | **5** |
| **11** | The use of telehealth services mitigates some time toxicity associated with receiving care for advanced cancer. | **0** | **1** | **6** | **55** | **39** | **4** |
| **12** | Patients with advanced cancer who are trusting of the healthcare system may experience less time toxicity. | **4** | **20** | **26** | **39** | **11** | **4** |
| **13** | In my experience, concerns about treatment time frequently affect patients’ decisions to discontinue treatment | **0** | **53** | **20** | **27** | **0** | **2** |
| **14** | (Open ended question) Are there any factors that we haven’t asked about that *you* think contribute significantly to the ‘toxicity’ of time spent coordinating and receiving treatment? | | | | | |  |
|  | **Question** | Strongly disagree (1) | Disagree (2) | Neutral (3) | Agree (4) | Strongly Agree (5) |  |
| **15** | Time spent pursuing treatment may prevent patients with advanced cancer from achieving other important goals. | **0** | **9** | **35** | **46** | **9** | **4** |
| **16** | It is challenging to determine if treatment time is truly perceived as ‘toxic time’ by patients with advanced cancer. | **0** | **6** | **11** | **66** | **17** | **4** |
| **17** | Patients frequently express concerns about the time required for palliative systemic treatments. | **1** | **59** | **26** | **12** | **2** | **2** |
| **18** | Oncologists can do a lot to modify healthcare contact time for patients with advanced cancer | **2** | **11** | **31** | **47** | **9** | **4** |
| **19** | I frequently discuss the time costs of treatment with patients with advanced cancer | 1 | 11 | 19 | 58 | **11** | **4** |
| **20** | Discussing treatment time helps patients with advanced cancer anticipate all of the relevant costs of care. | **1** | **4** | **16** | **65** | **15** | **4** |
| **21** | I have sufficient information at hand to disclose the time costs of palliative systemic treatments | **6** | **31** | **18** | **39** | **6** | **3** |
| **22** | It is difficult to discuss the time costs of care without causing information overload. | **0** | **29** | **18** | **39** | **15** | **4** |
|  | **Instruction** | Consider your day-to-day practice caring for patients with advanced cancer | | | | |  |
| **23** | I emphasise treatment time more in these patient groups | Frail/elderly | | | | |  |
|  |  | Poor performance status | | | | |  |
|  |  | High symptom burden | | | | |  |
|  |  | Socially isolated | | | | |  |
|  |  | Geographically isolated | | | | |  |
|  |  | Treatment benefit perceived to be marginal | | | | |  |
|  |  | None (I discuss treatment time equally with all patients) | | | | |  |
|  | **Question** | Strongly disagree (1) | Disagree (2) | Neutral (3) | Agree (4) | Strongly Agree (5) |  |
| **24** | Time spent receiving treatment is an important consideration when discussing treatments with patients with advanced cancer | **0** | **3** | **8** | **69** | **19** | **4** |
| **25** | I consider the impact of treatment time when recommending treatment options | **0** | **9** | **22** | **55** | **14** | **4** |
| **26** | Treatment efficacy and safety are more important than treatment time or logistics in treatment decision-making | **1** | **14** | **28** | **44** | **13** | **4** |
| **27** | (Open ended question) How do you know whether treatment time is ‘toxic’ time for patients | | | | | | N/A |
| **28** | (Open ended question) Do you have any additional comments or insights into the concept of time toxicity in cancer treatment | | | | | | N/A |
